# Silver Nanoparticles Alter Microtubule Arrangement, Dynamics and Stress Phytohormone Levels

**DOI:** 10.3390/plants11030313

**Published:** 2022-01-25

**Authors:** Jindřiška Angelini, Ruslan Klassen, Jitka Široká, Ondřej Novák, Kamil Záruba, Jakub Siegel, Zuzana Novotná, Olga Valentová

**Affiliations:** 1 Department of Biochemistry and Microbiology, University of Chemistry and Technology Prague, Technická 3, 166 28 Prague, Czech Republic; qwerty041195@gmail.com (R.K.); novotnaz@vscht.cz (Z.N.); valentoo@vscht.cz (O.V.); 2 Laboratory of Growth Regulators, Institute of Experimental Botany of the Czech Academy of Sciences & Faculty of Science of Palacký University, Šlechtitelů 27, 78371 Olomouc, Czech Republic; jitka.siroka@upol.cz (J.Š.); ondrej.novak@upol.cz (O.N.); 3 Deparment of Analytical Chemistry, University of Chemistry and Technology Prague, Technická 3, 166 28 Prague, Czech Republic; kamil.zaruba@vscht.cz; 4 Department of Solid State Engineering, University of Chemistry and Technology Prague, Technická 3, 166 28 Prague, Czech Republic; siegelj@vscht.cz

**Keywords:** *Arabidopsis thaliana* cotyledon, silver nanoparticles, silver ion, microtubular pattern, microtubular dynamics, stress phytohormones, jasmonic acid, abscisic acid, FRAP method, *gl-1* mutant

## Abstract

The superior properties of silver nanoparticles (AgNPs) has resulted in their broad utilization worldwide, but also the risk of irreversible environment infestation. The plant cuticle and cell wall can trap a large part of the nanoparticles and thus protect the internal cell structures, where the cytoskeleton, for example, reacts very quickly to the threat, and defense signaling is subsequently triggered. We therefore used not only wild-type *Arabidopsis* seedlings, but also the *glabra 1* mutant, which has a different composition of the cuticle. Both lines had GFP-labeled microtubules (MTs), allowing us to observe their arrangement. To quantify MT dynamics, we developed a new microscopic method based on the FRAP technique. The number and growth rate of MTs decreased significantly after AgNPs, similarly in both lines. However, the layer above the plasma membrane thickened significantly in wild-type plants. The levels of three major stress phytohormone derivatives—jasmonic, abscisic, and salicylic acids—after AgNP (with concomitant Ag^+^) treatment increased significantly (particularly in mutant plants) and to some extent resembled the plant response after mechanical stress. The profile of phytohormones helped us to estimate the mechanism of response to AgNPs and also to understand the broader physiological context of the observed changes in MT structure and dynamics.

## 1. Introduction

Nanotechnology has been one of the most rapidly advancing fields of human activity in the past decade. The unsurpassed physical and chemical properties of nanoparticles (NPs) and their relatively low cost have promoted their infiltration into each area of human life. The high stability of, e.g., metal NPs has resulted in their low biodegradability and potential high risk for ecosystems in the future. Some reports have described the benefits of NPs for plants. Engineered nanovectors can deliver bioactive compounds encapsulated in biopolymers inside cells in the plant body, for example [1]. Carbon, zinc oxide, titanium oxide, copper oxide, or even silver nanoparticles (AgNPs) can improve the germination, growth rate, biomass accumulation, and root elongation of many model plants and crops, usually at very low concentrations in soil or growth medium, as reviewed by Siddiqui et al. [2]. In fact, the most frequently mentioned toxic effects on plants may depend on the composition, stability, size, shape, or coating of the NP [3] and on the plant species; the electrochemical charge of NPs also influences the result, but in general, higher concentrations and a decrease in NP size cause more damage to plants [2,4,5,6].

Engineered NPs represent entirely new and unknown entities for plants; the most widely used in the world are AgNPs—25% of all consumer products [4]. The highest concentration of AgNPs recorded in contaminated water was 145 mg/L [7]. The size range of NPs is between 1 and 100 nm and, in the case of AgNPs, can contain 20–15,000 atoms. AgNPs oxidized in a humid environment can turn into more dangerous silver ions (Ag^+^) [8]. The negative effect of AgNPs on plant physiology is generally described as reduced root and shoot length, reduced number of root hairs, inhibition of leaf expansion and photosynthetic efficiency, reduced chlorophyll content, Ca^2+^ and reactive oxygen species (ROS) induced in the cytoplasm and lipid peroxidation [4,5,6].

Various barriers on the surface of the plant protect the inner cellular structures from the penetration of particles or chemical compounds [9]. How many NPs penetrate the plant body depends mainly on these primary barriers: the plant cuticle and cell wall [10,11,12], thus all subsequent signaling events and defense responses also depend on these barriers. The plant cuticle is the outer layer that covers the aerial parts, roots, and recently, a modified cuticle was found on the root cap of *Arabidopsis* [13]. Hydrophobic molecules can accumulate in/on the cuticle placed above the anticlinal cell walls and penetrate the apoplast along the middle lamellae [12]. The *Arabidopsis* trichomes are covered with a cuticle that comprises relatively large amounts of higher alkanes (C_32+_) compared to the cuticle of pavement cells [14]. A different micro-relief of the cuticle on stem pavement cells and the surface of trichomes were reported as well [15]. For example, mutant *gl-1 (glabra-1)* plants lack trichomes on stems and leaves (TAIR database; www.arabidopsis.org); in addition, some authors reported a significantly lower level of alcohols and alkanes in mutant *gl-1* cuticular waxes [16], while other authors attribute changes in the composition of the cuticular waxes of the mutant *gl-1* purely to the absence of trichomes. Thus, a different uptake of NPs in the wild-type and mutant *gl-1* plants used in all of our experiments would be expected. Our preliminary results showed a different cotyledon cell surface micro-relief in wild-type and *gl-1* mutant plants (Figure S1). *GL-1* (*GLABRA-1*: At3g27920) is the gene coding a transcription factor, also called MYB0. After GL-1 interaction with JAZ (jasmonate zim domain) and two of the five DELLA proteins present in *Arabidopsis* (switching between gibberellic and jasmonic acid signaling), trichome initiation is regulated. Therefore, GL-1 is involved in gibberellic or jasmonic acid-mediated signaling [17]. Moreover, the proper function of MT and microtubule-associated proteins (e.g., the ZWI gene encodes the motor protein kinesin—downstream of GL-1) is essential for trichome initiation [18,19]. In cooperation with other MAPs, it contributes to cell wall-MT crosstalk and cell wall formation in epidermal cells [20,21,22].

The cell wall, located between the cuticle and the plasma membrane, contains a fibrous and amorphous network of various polysaccharides that forms a sieve-like barrier that is effective against particles larger than approximately 20 nm. At the same time, the cell wall can act as a reservoir for various NPs [6,8]. Treatment with negatively charged gold NPs (but not positively or neutrally charged) for 7 days caused a thickening of the outer rhizodermal cell wall in the meristematic zone of the *Arabidopsis* root. Thickening of the outer tangential cell wall has also been described as an effect of heavy metals (Al, Pb, Cd) [23]. AgNPs as large as 80 nm were accumulated in *Arabidopsis* root cap cells, more in axial than tangential cell walls, in the middle lamella, and AgNP clusters blocked transport through the plasmodesmata [6]. The reported cell wall pore sizes vary slightly across the literature, depending on the author and the methods used for detection. However, different NP sizes ranging from 5 to 20 nm have been detected within the cell walls of different plants [24].

The number of perceived stress signals in the plant body leads to particular changes in the phytohormonal profile, which integrates signals and promotes a specific plant response to stress event/events, such as NPs. Various biotic and abiotic stress conditions trigger an elevated level of phytohormones, but the final plant response is orchestrated together with specific changes in the profiles of other hormones in individual plant tissues or organs [25]. The level of particular phytohormones in connection with a specific stress (e.g., wounding) has its own dynamics over time [26,27]. Gibberellic acid and cytokinins are well-known essential phytohormones that alleviate the stress caused by heavy metals, but another phytohormone (e.g., abscisic acid, auxin brassinosteroid, and ethylene) participates in carrying out the unique plant response necessary upon encountering heavy metal toxicity [3]. After the heavy metal’s action and subsequent ROS induction, ABA increased to close the stomata, decreased transpiration flow, and thus decreased metal expansion from the root to the aerial parts of the plant. Heavy metals decrease the level of endogenous auxin, leading to a reduced root meristem size and suppressed primary root elongation. The exogenous application of brassinosteroids prior to heavy metals improved antioxidant system activity and enhanced plant growth under stress conditions. Furthermore, plants increase the level of ethylene after heavy metal treatment, which reduces plant growth and development and facilitates plant acclimation to stress [25,28]. For these reasons, stress-related phytohormones can be used as natural markers to monitor the effect of NPs on plant growth and development.

Despite relatively low NP internalization and limited long-distance movement, the plant can perceive the presence of NP and develops a defense response at many subcellular or organ levels simultaneously [29,30]. According to the nature of the metal NPs, we can assume mechanical damage to plant cell structures, especially when NPs create clusters within the plant body (e.g., silver itself has a tenfold higher density than the cytosol) and heavy metal stress is reported (ions released from NPs) [31,32]. NPs can bind to a wide range of proteins and thus impair protein function [4]. The self-organization of microtubules (MTs) represents one of a few actively mobile parts of the plant body and predetermines MTs to participate primarily in maintaining cell morphology, in cell division, and in promoting cell wall development [33]; moreover, MTs are essential for intracellular transport [34]. Fast growth and MT disassembly (hundreds of nanometers per second) is the function of MTs themselves, but the formation of a complicated microtubular net inside the cells and the dynamics of this process are modulated and controlled by a variety of microtubule-associated proteins (MAPs) [35,36]. A dense network of MTs beneath the plasma membrane performs a sensoric function under stress conditions [37]. Remodeling the cytoskeletal architecture is one of the first plant responses to most stresses, and it can trigger many other signaling events, leading plants to effective defense [38,39]. The right timing of cytoskeleton rearrangement or de/polymerization in individual plant cell types creates an essential plasticity, which facilitates plant acclimation to external conditions, for example to salt stress in *Arabidopsis* plants [40]. MT depolymerization is part of the stimulation of calcium channels, which are a basic component of the early response to most stresses [41]; moreover, MT depolymerization could be an early sign of programmed cell death (PCD) [42]. Mechanical pressure caused by a fungal appressorium or needle was perceived in *Arabidopsis* epidermal MTs within a few minutes by extensive reorganization and bundling of the MT at the irritated location [39]. Furthermore, neighboring cells react by creating ‘continuous’ supracellular patterns around the mechanically stimulated cell [43].

In this study, we focused on understanding the stress response caused by AgNPs or the Ag^+^. We hypothesized different levels of AgNP uptake in epidermal cells and in trichomes. To reveal this mechanism, at least in part, in addition to wild-type plants, in all experiments we also used *gl-1* mutant plants that lack trichomes, and in which a different composition of cuticular waxes on the cell surface has been described. Both plant lines had GFP-labeled microtubules.

The first objective of this research was to compare the effect of treatments on the abundance of the major stress-related phytohormones and their derivatives (precursors and metabolites). Changes in phytohormone profiles may indicate a defense mechanism by which the plant copes with stress caused by AgNPs. Our next objective was to determine whether AgNPs/Ag^+^ affect MT structure and MT dynamics, which respond rapidly and sensitively to many applied stresses. MT dynamics was evaluated using an innovative method based on the FRAP technique performed in a confocal laser scanning microscope. In addition, changes in layer above plasma membrane was also studied to test the hypothesis that AgNPs affect cell wall thickness through MT dynamics. We hypothesize that AgNPs in dynamic equilibrium with Ag^+^ can induce mechanical stress or heavy metal-induced stress in plant cells. The results obtained are discussed from a broader cytological and physiological perspective.

## 2. Results

### 2.1. Direct Visualization of AgNPs Clusters and Specific Changes in GFP-TUA6 Arabidopsis

**Figure 1:**
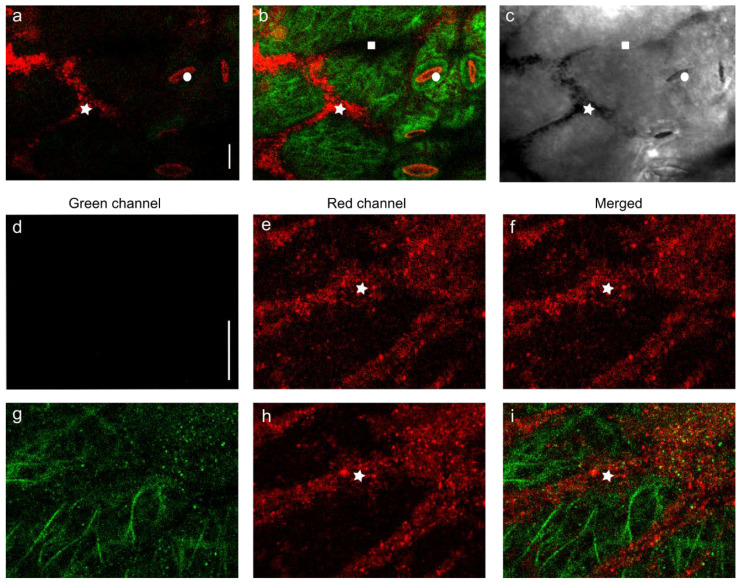
Clumps visualized in *Arabidopsis* cells after AgNPs treatment. 5-day-old plants with GFP-tagged microtubules (GFP-TUA6) were treated for 24 h with 150 mg/L AgNP solution. The images were taken sequentially with a Zeiss 880 CLSM in the green channel (ex/em: 488/496–556 nm) for GFP visualization—(**d**,**g**), or in the red channel (ex/em: 561/563–652 nm) for AgNPs visualization—(**e**,**h**); the pictures (**a**,**b**,**f**,**i**) are the merge of both channels. Picture (**c**) represents the DIC image of picture (**b**). The first row of pictures shows cotyledon epidermal cells; (**a**) focuses on the surface of epidermal cells, (**b**,**c**) is the optical layer 1.6 µm under (**a**)—inside the region with a cortical MT. The white asterisks indicate the deposition of an AgNP clump (red or black—DIC) on the surface of grooves between the cells. Squares indicate grooves between the cells without an AgNP deposition. The circles indicate a stomatal pore with accumulated AgNPs. The second row of images shows details of AgNP clusters on the surface of epidermal cells. The third row of pictures shows the optical layer 1 µm below the second—immediately beneath the plasma membrane in the outer part of the microtubular layer; the green spots in the image (**g**) represent clumps of free tubulin units tagged with GFP. Scale bars = 10 µm.

Over time, direct exposure to the AgNP solution caused an increasingly intense brown coloration visible to the naked eye on *Arabidopsis* surface tissues. Confocal laser scanning microscope (CLSM) with differential interference contrast (DIC) imaging allowed us to observe the detailed localization of AgNPs. The resolution of the confocal microscope is over 200 nm at both wavelengths of excitation light used, so it was possible to see red or black clusters of AgNPs (Figure 1) but not the nanoparticles themselves, which are 20 nm in size. Epidermal cells were discontinuously covered with AgNPs clustered on the surface of the cell wall and in grooves between adjacent cells, but also accumulated in the stomatal pores over time (Figure 1a–c—red color). Clumps of AgNPs were located on the cuticle surface, in the cell wall, and also under the plasma membrane (Figure 1d–i and Figure S2j–s); these clumps were not observed in plants treated with growth medium (Figure S2a–i). The process proceeded similarly in both cotyledons and hypocotyls (Figure 1 and Figure S2). No differences in AgNP accumulation were observed between the two plant lines used (WT, *gl-1* mutant). CLSM allows precise orientation on the Z axis (in the direction from the surface to the interior of the cell) if an invariant reference point is chosen. Our landmark was the location of the MT array right under the plasma membrane, where the MT appeared. MT in both plant lines were labeled with GFP and formed a layer approximately 1 μm wide under the plasma membrane. Furthermore, in AgNPs-treated plants, fluorescent green clumps that did not differ in shape from the red clumps of AgNP were also observed in the region where the MT occurred, just below the plasma membrane, (Figure 1g,i).

### 2.2. Hormonal Profiles in Whole Arabidopsis Plants after AgNPs and Ag^+^ Treatment

Plant hormones are crucial signaling molecules that coordinate all aspects of plant growth, development, and defense. A number of perceived stress signals in the plant body lead to particular changes in the phytohormonal profile, which integrates signals and promotes a specific plant response to a stress event/events. We investigated whether AgNPs could affect the level of plant phytohormones. Three main stress-related phytohormones (JA, ABA and SA) and their derivatives were measured in AgNP-treated plants. We compared the *Arabidopsis* wild-type with the mutant line *gl-1*.

The levels of nearly all derivatives of JA, ABA, and SA increased significantly after 6 h of treatment with 150 mg/L AgNPs compared to the application of 50 μg/L of Ag^+^ or non-treated plants. The non-treated *gl-1* line had significantly increased levels of JA (1.4-fold) as well as its precursor (*cis*-(+)-12-oxo-phytodienoic acid *(cis*-OPDA, 2.5-fold) and hydroxy-JA catabolites (11,12-hydroxy-JA, 2.3 times) compared to the WT (Table 1: Blank). Interestingly, the active form, (-)-jasmonoyl-L-isoleucine (JA-Ile) did not differ significantly between lines after control treatment; moreover, the level of catabolite (±)-9,10-dihydrojasmonic acid (9,10-DHJA) was even lower in the mutant plants.

Compared to non-treated WT plants, the level of *cis*-OPDA increased approximately 4.8-fold after treatment with Ag^+^ and remained similar after AgNPs. Surprisingly, the level of *cis*-OPDA decreased significantly (2.1-fold) in *gl-1* mutant plants after Ag^+^ treatment, however, a significant increase (1.8-fold) was recorded after AgNPs compared to non-treated plants. A similar pattern was observed in mutant plants for the levels of JA catabolites, 9,10-DHJA and 11-OH-JA/12-OH-JA as follows: a decrease (6.2-fold) after Ag^+^ treatment and a significant increase (9.7-fold) after AgNPs treatment compared to ion treatment. While the JA catabolite levels in the WT line grew gradually (blank >> Ag+ >> AgNPs), only the concentration of hydroxy-JA catabolites increased significantly (4.5-fold) after AgNPs compared to ion treatment. The level of active JA forms (JA and JA-Ile) in both lines gradually increased (blank >> Ag+ >> AgNPs). The highest level increase of all after AgNPs compared to Ag^+^ treatment was exhibited by an active JA form, JA-Ile (WT: 13.1×, *gl-1*: 14.7×) in both lines. Importantly, JA-Ile was significantly (1.9 times) higher in mutant *gl-1* plants compared to the WT (Table 1). In summary, our results of jasmonate profiling showed that the effect of stress factors (e.g., cell wall damage) on the levels of JA and its metabolites induced by AgNPs treatment was higher than for Ag^+^ treatment.

Abscisic acid and two of its main metabolites: phaseic acid (PA) and dihydrophaseic acid (DPA) elevated their levels after treatment with AgNPs (Table 2). In both lines, increases in ABA levels were measured after AgNP treatment compared to Ag^+^ treatment (WT: 7.9-fold, gl-1: 16.9-fold). Moreover, similar significant differences between the lines were observed after control treatment (non-treated plants: WT 2.0 times lower than *gl-1*) and after AgNPs treatment (WT 2.3 times lower than *gl-1*) (Table 2). Interestingly, DPA and PA were not detected after the control treatment, nor after the treatment with Ag^+^. PA levels rose significantly after AgNPs in WT and *gl-1* plants without significant differences between the lines. Similarly, the level of DPA was detectable only 6 h after AgNPs treatment, however, WT plants and mutant *gl-1* plants differed significantly. In summary, AgNPs significantly affect abscisate levels, which again confirms their possible negative effect on the development of mutant *gl-1* plants.

The last phytohormone, salicylic acid, was also affected by AgNPs. In WT plants, SA was only significantly elevated (1.5-fold) when comparing the control to AgNPs treatment. The level of SA in mutant plants followed the trend (blank > Ag^+^ > AgNPs) similarly to that of ABA and some JA derivatives, a significant decrease was observed after ion treatment and a steep increase in SA level occurred after AgNPs. Plant lines differed after Ag^+^ treatment (WT 2.2 times higher than gl-1) and after AgNPs (WT 1.8 times lower than gl-1).

### 2.3. Changes in Microtubule Pattern after Treatment with Ag^+^ or AgNPs

Significant changes in the levels of all stress hormones were observed after AgNP treatment, and similarly the responses of the whole plants differed significantly between the WT and *gl-1* lines. Therefore, we were interested in how changes in hormone levels manifested themselves at the subcellular level. We investigated the pattern of cortical microtubules in cotyledon epidermal cells. In particular, a random or parallel arrangement of MTs, their shape, and additional fluorescent structures that emerge after particular treatments and times were observed.

**Figure 2:**
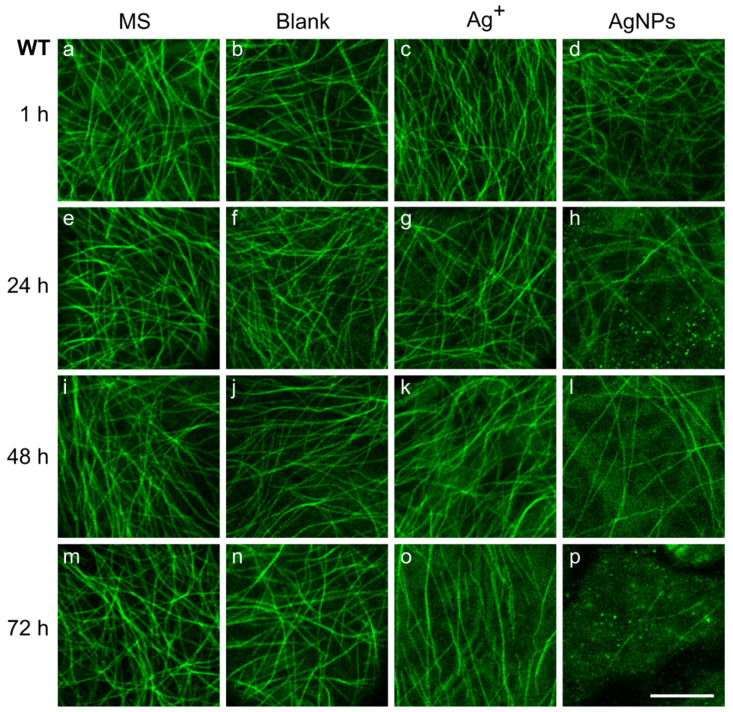
Pattern of cortical microtubules in wild-type cotyledon pavement cells. 7-day-old *Arabidopsis* **WT** plants were treated for 1 (**a**–**d**), 24 (**e**–**h**), 48 (**i**–**l**), or 72 h (**m**–**p**) with growth medium (MS) (**a**,**e**,**i**,**m**), nanoparticle stabilizing buffer (Blank) (**b**,**f**,**j**,**n**), 50 μg/L Ag^+^ (**c**,**g**,**k**,**o**) or 150 mg/L AgNPs (**d**,**h**,**l**,**p**) solution. Images represent one optical section (optimal axial resolution: 380 nm) of the central part of the cell acquired with a Zeiss 880 CLSM, objective: Plan-Apochromat 63x/1,4 Oil DIC M27. Plants had a tubulin α6 subunit tagged with GFP for microtubule visualization (ex/em: 488/516–561 nm). Scale bar = 10 µm.

**Figure 3:**
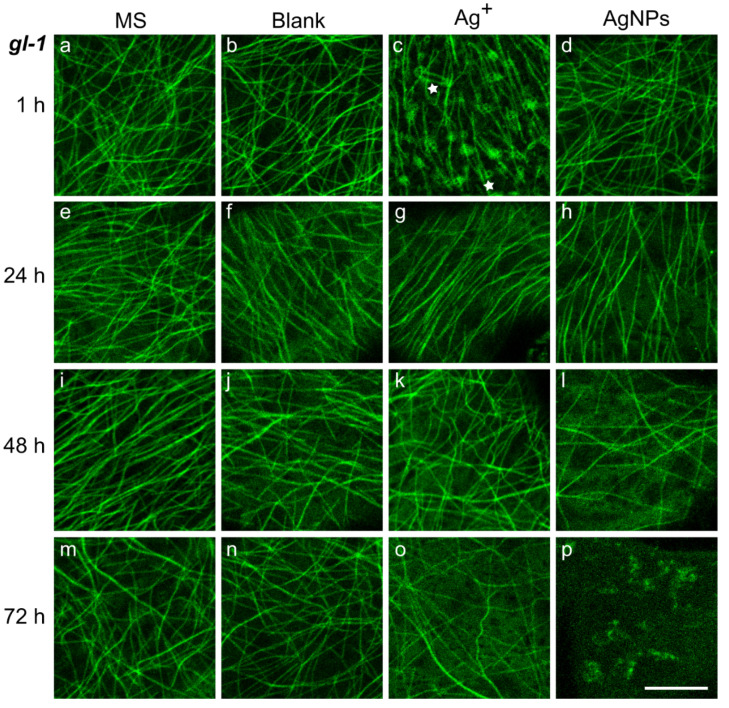
Pattern of cortical microtubules in *gl-1* mutant cotyledon pavement cells. 7-day-old *Arabidopsis **gl-1*** mutant plants were treated for 1 (**a**–**d**), 24 (**e**–**h**), 48 (**i**–**l**), or 72 h (**m**–**p**) with growth medium (MS) (**a**,**e**,**i**,**m**), nanoparticle stabilizing buffer (Blank) (**b**,**f**,**j**,**n**), 50 μg/L Ag^+^ (**c**,**g**,**k**,**o**) or 150 mg/L AgNPs (**d**,**h**,**l**,**p**) solution. The upper asterisk (in image c) is placed below the lens-shaped particle surrounded by the fluorescence signal, whereas the lower asterisk is located below the dot-shaped particle surrounded by the fluorescent circle. Images represent one optical section (optimal axial resolution: 380 nm) of the central part of the cell acquired with a Zeiss 880 CLSM, objective: Plan-Apochromat 63×/1,4 Oil DIC M27. Plants had a tubulin α6 subunit tagged with GFP for microtubule visualization (ex/em: 488/516–561 nm). Scale bar = 10 µm.

In plants treated with growth MS medium, the cortical MTs of pavement cells were arranged relatively randomly, in both WT and *gl-1* lines (Figure 2 and Figure 3a,e,i,m). It was only in mutant *gl-1* plants 1 h and 48 h after MS treatment that almost identical numbers of cells were counted with a random or parallel arrangement of MTs. Thicker and more luminous filaments were sometimes observed among other MTs in both lines; they were bundles of several MTs. In general, randomly-arranged MTs were long, sometimes bent into an arc (Figure 2a,e). The MTs in a parallel arrangement were occasionally a little wavy (1–3 waves per microtubule) (Figure 2i). The described MT pattern is considered to be characteristic for non-stressed WT plants [44]. Some observed cells had two different parts in terms of the arrangement of MT, one half was random, the second in parallel arrays (e.g., Figure 3e).

The blank treatment was used as a control treatment for the AgNP solution. The blank is a mixture of 1/16 MS and stabilizing buffer for AgNPs (2:1). The arrangement of MT resembled the pattern observed after growth medium at almost all time points and for both lines. The random MT order predominated over the parallel arrangement. Only mutant plants 24 h after blank treatment had a higher number of cells with parallel MT organization compared to the rest of the time points. A lower density of MT was discernible 72 h after treatment in both lines (Figure 2 and Figure 3n). The plants of both lines had a few wavy microtubules in some cells, but compared to the waves observed after MS, these MT looked more corrugated, with 5–6 waves in the MT (see e.g., Figure 2f). In approximately one third of the cells, bundles of several MT (usually with a higher bright intensity) were observed (e.g., Figure 3b,j).

Silver ions (Ag^+^—50 µg/L) are inherently present in the solution with AgNPs; therefore, the solution of them was used as another control for the AgNPs treatment. The MT pattern in the presence of Ag^+^ quite often exhibited MT bundles, and very often a wavy arrangement in both lines 1 h after treatment (Figure 2c). MTs were placed mostly in parallel. Furthermore, among the MTs in mutant *gl-1* plants, there were visible black points with a circular or irregular fluorescent signal around them. Round-shaped regular circles had quite a uniform size, approximately 750–850 nm in diameter with a fluorescent ring around inner black dots 450–550 nm in diameter (Figure 3c, marked with a lower asterisk). Sometimes mobile, lens-shaped, around 5- to 10-µm-long particles (tens per cell) surrounded by a fluorescent signal were observed (Figure 3c, marked with an upper asterisk). Simultaneously, detailed visualization with differential interference contrast revealed a correlation with tiny dots and lens-shaped organelles of identical sizes in the cytosol (Figure S3). In the WT cotyledons, 1 h after Ag^+^, MTs were mostly arranged in a parallel organization without the additional structures seen in mutant plants. After 24 h of Ag^+^ action, MTs of both lines were in parallel or random order without additional structures; the wavy MTs were relatively rare (Figure 2 and Figure 3g). However, longer (48 and 72 h) treatment with Ag^+^ induced more wavy and bundled, parallel MTs, with mutant plants being influenced to a higher extent. After 48 and 72 h, no other structures were detected among the MTs. Nevertheless, the MTs seemed to float in a fluorescent fog, the background was not as black as before. A more highly fluorescent background was usually associated with a visibly smaller amount of MT (Figure 2 and Figure 3k,o).

AgNPs caused obvious damage in plant MTs over time. MTs in cotyledon epidermal cells were relatively randomly ordered in both lines and at all time points, black regular circles near the MTs were sometimes present 24 or 48 h after AgNPs treatment (Figure 2 and Figure 3h,l). Furthermore, there was often a noticeable diffuse fluorescence signal in the background with bright spots (250–500 nm in diameter) observed more frequently in the WT line. (Figure 2h,p). The treatment 48 h after AgNPs caused further progression of MT depolymerization; sparser microtubular network was observed compared to the other treatments and to the 1 and 24 h AgNPs treatments. In addition, shorter, straight, and rod-shaped MTs were observed. A stronger fluorescence background was observed in almost all observed cells of both lines 48 h after AgNPs (Figure 3l), but bright spots were even smaller than in the images taken 24 or 72 h after NPs treatment (Figure 2h,p). A few or no MTs were noticed 72 h after AgNP treatment in both lines; a diffuse fluorescence signal was often observed, sometimes with black bodies (regular circles (450–550 nm in diameter) or lens-shaped 5 to 10 µm long particles) that did not move. The cotyledon MTs after AgNPs were much less wavy than after Ag^+^ at most time points after treatment (Figure 2 and Figure 3p).

### 2.4. The Number of MTs Appearing De Novo in Cotyledon Cells

Images of the microtubule patterns revealed various changes in the cortical layer of the epidermal cell cytoplasm of cotyledons.

To precisely evaluate the changes in MT occurrence under the same conditions as described above, the number of MTs was measured for a randomly selected area in the cortical layer of cotyledon cells. For one cell, usually up to five regions with the same size were chosen.

**Figure 4:**
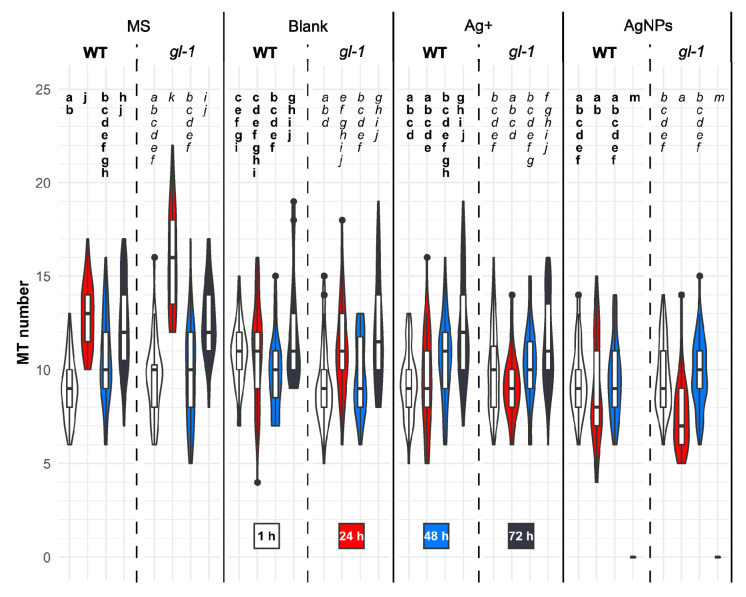
Number of de novo-raised microtubules in specified regions of cotyledon epidermal cells. 7-day-old *Arabidopsis* plants (wild-type or mutant *gl-1*) were treated for 1, 24, 48, or 72 h with growth medium (MS), nanoparticle stabilizing buffer (Blank), 50 µg/L Ag^+^ or 150 mg/L AgNPs. Images of one optical section (optimal axial resolution: 380 nm) were acquired with a Zeiss 880 CLSM and analyzed with the software ImageJ. Values represent averages of de novo-arising MT ± SD demonstrated with white boxes inside the violin plot area (*n* = 35–100). The width of the violin plot represents the number of observations counted for an individual time point after treatment. Significance is determined by multifactorial ANOVA and the post hoc unequal Tukey test. Different letters denote significant differences at *p* < 0.05.

In conditions without stress factors, after growth medium treatment, a fluctuating number of MTs that appeared de novo in the observed area was perceived in both lines. One hour after treatment, on average nine MTs grew de novo in the observed region not only after growth medium, but also after all treatments. After MS treatment, a higher amount of raised MTs was usually observed in mutant plants. Twenty-four hours after MS treatment, the difference was significantly higher in *gl-1* plants compared to the WT; after 48 h the amount decreased dramatically, and 72 h after treatment the amount increased again in both lines (Figure 4: MS treatment).

A mixture of 1/16 MS and NP stabilization buffer (2:1)-blank significantly reduced the incidence of new MTs compared to growth medium treatment after 24 h in both lines. The time course of the MT number values was similar to the MS treatment. Only wild-type plants treated with the blank for 1 h had a significantly higher number of de novo-emerged MTs compared to the mutant plants and plants of both lines treated with MS for 1 h (Figure 4: blank treatment). Ag^+^ caused a slow increase in newly raised MTs over time, but there were no significant differences between lines. The number of de novo-emerged MTs after Ag^+^ did not differ significantly compared to MS treatment after 1, 48, and 72 h, but the amount of MTs was significantly lower 24 h after Ag^+^ in both lines.

Interestingly, the number of de novo-raised MTs increased or decreased over time in an opposite trend to that of the values of the area occupied by the MTs. This phenomenon was observed in both lines after treatment with MS, blank, and Ag^+^; unlike treatment with AgNPs both de novo-appeared MT and the area occupied by MT exhibited a similar trend over time (with the exception of after 48 h, Figure 4 and S4).

AgNPs significantly reduced the number of newly emerged MTs after 24 and 72 h compared to the blank control in both lines. However, the values did not vary significantly between wild-type and mutant *gl-1* lines. AgNPs caused a decrease in de novo-formed MTs after 24 h, followed by an increase (48 h), and finally, depolymerization of the MT network was almost complete 72 h after treatment, and no MTs appeared de novo at that time. (Figure 4, AgNPs)

### 2.5. AgNPs Differently Decelerate Microtubular Dynamics in WT and Mutant Plants

**Figure 5:**
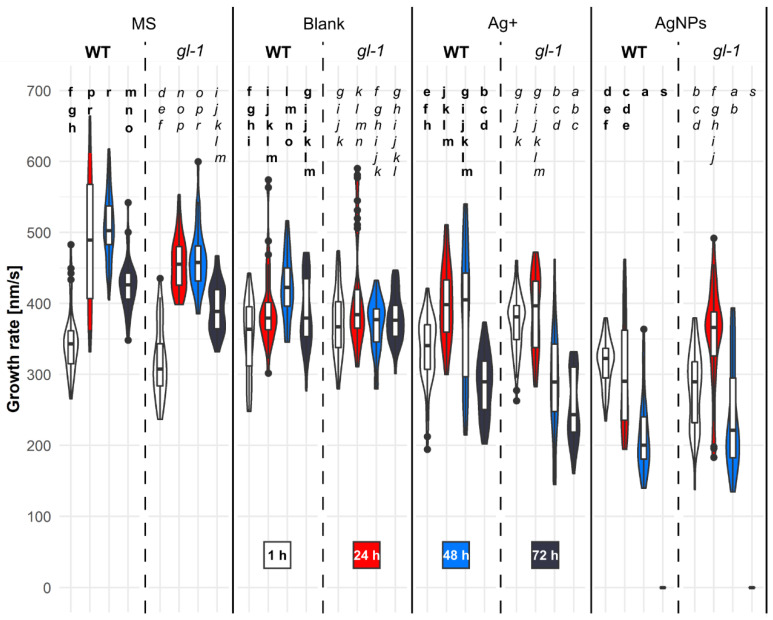
Growth rate of de novo-emerged microtubules in specific regions of cotyledon epidermal cells. Seven-day-old *Arabidopsis* plants (wild-type or mutant *gl-1*) were treated for up to 1 h, for 24, 48, or 72 h with growth medium (MS), nanoparticle stabilizing buffer (blank), 50 µg/L of silver ions (Ag^+^), or 150 mg/L of silver nanoparticles (AgNPs). The time stacks of images (optimal axial resolution: 380 nm) were acquired with a Zeiss 880 CLSM and analyzed with the software ImageJ after creating kymogram. Values represent averages of the growth rate of de novo-arising MT ± SD, shown by white boxes inside the violin plot area (*n* = 35–100). The width of the violin plot represents the number of observations seen for the individual speed of microtubules. Significance is determined by multifactorial ANOVA and the post hoc unequal Tukey test. Different letters denote significant differences at *p* < 0.05.

The MT de novo growth rate in unstressed plants varied nonsignificantly over time in both wild-type and mutant plants; growth rate increased constantly up to 48 h, then decreased (Figure 5, MS).

The mixture of 1/16 MS and stabilizing buffer for nanoparticles (2:1)—the blank—up to 1 h augmented the speed of MT growth compared to MS treatment, and significantly in mutant plants. The course of the growth rate curve over time in wild-type plants resembled those treated with MS—the maximum speed was observed 48 h after treatment, unlike mutant *gl-1* plants, which had the highest growth rate 24 h after blank treatment (Figure 5). At the other time points, the growth rate of MTs in both lines was slower than after MS treatment (WT 24 and 48 h, *gl-1* 48 h—significantly).

A 50 μg/L solution of Ag^+^ significantly reduced MT growth rate at all time points in WT plants and, except for the 1 h treatment, also in mutant plants. In contrast, mutant *gl-1* plants exhibited a significantly higher MT growth rate 1 h after Ag+ treatment compared to MS treatment. The trend of the speed curve was similar for both lines to that after MS treatment (Figure 5: Ag^+^ vs. MS).

AgNPs (150 mg/L) with inherent Ag^+^ (50 µg/L) reduced the microtubular growth rate at all time points compared to the other treatments. Compared to the blank, a significant decrease was observed at all time points for mutant plants, and for WT plants after 24, 48, and 72 h. AgNPs significantly reduced the MT speed in WT plants 24, 48, and 72 h after treatment compared to Ag^+^.

MTs in *gl-1* mutant plants significantly decreased their growth rate only 1 h and 72 h after AgNPs treatment compared to Ag^+^. Over time, there was a significant difference between lines 24 h after AgNPs treatment; WT plants consistently reduced the MT growth rate, but the *gl-1* line showed a transient increase in MT speed 24 h after treatment.

### 2.6. The Thickness of the Auramine O-Stained Layer in Cotyledons after Treatment with AgNPs

The cuticle and cell wall are the primary barrier that can significantly limit the entry of AgNPs into the cytosol of plant cells. To determine whether the thickness of the *Arabidopsis thaliana* cell wall changes during the treatment of AgNPs in wild-type and *gl-1* (*glabra1*) mutant plants as an enhanced plant protection, we visualized the cell wall and cuticle using Auramine O.

The fluorescent dye, Auramine O is capable of staining the plant cuticle and some parts of the primary or secondary cell wall, for example, suberin or lignin [45]. Both cutin and suberin contain polyesters of hydroxy fatty acids. Originally, Auramine O was a specific dye for the identification of *Mycobacterium tuberculosis*. The outer cell wall of *Mycobacterium* consists of some fatty acids called mycolic acid, the whole cell wall contains arabinogalactans, etc. [46]. Arabinogalactans are common compounds in plant cell walls. Auramine O was used for the specific staining of plant cuticle, e.g., [47], but our observations and previous information indicate that Auramine O stains all structures present above the plasma membrane in *Arabidopsis* seedlings, thus we were unable to distinguish a boundary between the epidermal cuticle and cell wall. We measured the layer above the plasma membrane as a whole, which was the only possibility for monitoring the difference in the thickness of the layer with a confocal microscope (due to the axial resolution limit of the microscope). Our preliminary results showed a different microrelief in WT and *gl-1* mutant plants (Figure S1). The layer visualized with Auramine O was placed above the plasma membrane of cotyledon epidermal cells. It was measured using confocal microscopy, ex/em: 458/491–571 nm and processed simultaneously with two independent methods.

**Figure 6:**
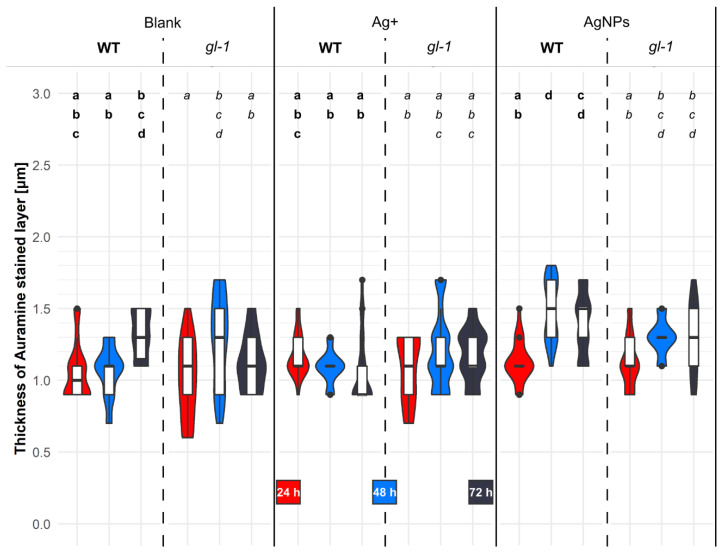
Thickness of extracellular layer stained with Auramine O in cotyledon epidermal cells. Five-day-old *Arabidopsis* plants (wild-type or mutant *gl-1*) were treated for 24, 48, or 72 h with nanoparticle stabilizing buffer (blank), 50 µg/L Ag+ or 150 mg/L AgNPs. Z-stack images (optimal axial resolution: 377 nm) were acquired with a Zeiss 880 CLSM and analyzed with the software ImageJ. Values represent averages of the thickness of the layer above the plasma membrane ± SD shown by white boxes within the violin plot area (*n* = 18–21). The width of the violin plot represents the number of observations seen for the individual time and treatment. Significance is determined by multifactorial ANOVA and the post hoc unequal Tukey test. Different letters denote significant differences at *p* < 0.05.

The thickness of the layer above the plasma membrane was checked 24, 48, and 72 h after treatment with a mixture of 1/16 MS and stabilizing buffer for AgNPs (2:1)—blank, Ag^+^—50 μg/L, and 150 mg/L solutions of AgNPs in which Ag^+^ were naturally present at the above-mentioned concentration. In this experiment, only the roots and part of the hypocotyl were treated with various solutions (Figure 6).

Twenty-four hours after each treatment in each line, the values of layer thickness were almost identical. Wild-type plants did not significantly change the thickness of the layer above the plasma membrane within that time after blank treatment. Forty-eight hours after treatment, the values slightly dropped, and after the next 24 h, the thickness of the Auramine O-stained layer increased by approximately 300 nm. Unlike mutant plants that had an opposite trend during this time, the values after 48 h increased significantly compared to the 24 h treatment and then decreased slightly again. No significant differences were observed between plant lines after any of the treatments or times (Figure 6: Blank).

The blank and Ag^+^ caused an inverted trend between lines over time. In wild-type plants, the thickness of the layer did not decrease significantly with time; in mutant plants, it increased.

Only AgNPs in wild-type cotyledon cells caused significant changes during the experiment; the thickness of the layer 48 h after treatment increased by about 370 nm compared to 24 h earlier. This value is also significantly higher than the thickness 48 h after treatment with blank or Ag^+^ ions. Treatment with AgNPs after 72 h caused some reduction in extracellular matter in the WT line, but still had a significantly higher value than after treatment with ions after 72 h. Mutant *gl-1* plants after AgNPs increased the thickness of the extracellular layer from 24 to 48 h by only about 170 nm, and remained the same for the next 24 h. Mutant plants had a similar thickness of the stained layer after all the treatments used at all the time points (Figure 6: AgNPs and Ag^+^).

## 3. Discussion

Imaging microtubular patterns at time points after treatment is a useful tool for the observation of long-term dynamics in the constantly changing MT network. Our bird‘s eye view of the situation revealed specific regularities for the chronology of MT damage caused by AgNPs or Ag^+^ treatment, as well as differences between *Arabidopsis* lines. All observed changes in MT arrangement were confirmed by quantifying MT dynamics. Together with the changes observed in the thickness of the layer above the plasma membrane, we tried to correlate these changes with changes in hormonal profiles and thus map the mechanism of plant defence after AgNPs treatment.

### 3.1. Nonstressed Plant Lines Differ in Microtubular Dynamics and Hormonal Profiles

Cortical MTs in cotyledon pavement cells were arranged in a relatively random organization, usually with a moderate degree of bundling under nonstress conditions, although a variable, low number of cells with parallel arrangements occurred over time (plants were grown in 1/16MS medium, under sterile conditions, without direct mechanical manipulation).

A cyclic reorientation of the cortical MT under the outer epidermal wall was described in elongating anisotropic cells, also in azuki or faba beans [48,49]. A process called bipolarization defined functionally different domains in the cell during the transition from a random to a parallel MT arrangement, in each half of the hypocotyl cell, MTs moving in opposite directions were detected [50]. In our experiments, non-stressed pavement cells with one half of the MTs randomly oriented and the other half in parallel were sometimes observed (Figure 2e). We can assume cyclic MT reorientation in growing pavement cells of *Arabidopsis* cotyledons as well, despite the fact that the direction of growth in lobular cells is not as obvious as in hypocotyl cells. This type of reorientation could be achieved by changes in MT dynamics (de novo MT nucleation, changes in MT rate growth, or the number of MTs in the cell).

A very high number of MTs that grew de novo (Figure 4), almost the highest growth rate observed (Figure 5), and at the same time a very low area occupied by MTs (Figure S4), were observed in both lines of plantlets 24 h after MS treatment. In *gl-1* mutant plants, the number of MTs were even higher than in the WT (the highest of all treatments, times and lines). Plants 24 h after treatment with 1/16MS corresponded to 8-day-old plants, under nonstressed conditions. The oscillations in MT dynamics described above reflect physiological changes in particular cells.

MT changes are also related to developmental processes: cell growth, cell wall thickening, etc. [33,51]. The sixth to eighth day of *Arabidopsis* growth is characterized by rapid development of the first true leaves [52]. Under nonstressed conditions, *gl-1* mutant plants expressed not only an exceptionally high number of de novo-growing MTs (Figure 4) (but not growth rate), but also a higher level of some JA derivatives, specifically 2.5 times higher *cis*-OPDA (Table 1) compared to the WT. Moreover, the phytohormonal control of trichome initiation in *Arabidopsis* was mediated, at least in part, by transcriptional regulation of the established TTG1 (TRANSPARENT TESTA GLABRA 1) complex and depends on the GLABRA 1 transcription factor [53]. After the interaction of GL-1 with JAZ (the jasmonate zim domain) and two of the five DELLA proteins present in *Arabidopsis* (switching between gibberellic and jasmonic acid signaling), trichome initiation is regulated. Therefore, GL-1 is involved in gibberellic or jasmonic acid-mediated signaling [17]. The significantly higher level of JA derivatives and MT number in mutant plants may be a kind of developmental compensation mechanism due to the lack of the GL-1 protein, and thus the inability to develop trichomes. The jasmonic acid (JA) signaling pathway is involved in the control of leaf growth; methyl jasmonate (MeJA) was described as a repressor of cell proliferation [54]. According to Flokova et al., in 24-day-old, untreated *Arabidopsis* WT leaves, almost identical levels of JA derivatives were detected at time 0 and after 24 h [27]. Induced JA signaling is inevitably associated with the inhibition of gibberellic acid (GA) signaling (GA promotes the growth of the plant) during the growth-versus-defense conflict. This switch to JA signaling is put into effect through DELLA proteins that compete with the MYC2 transcription factor for binding to the JAZ protein when GA is absent. Subsequently, free MYC2 activates the expression of JA-responsive genes [55]. In plants treated with Ag^+^ or AgNPs, right leaves developed significantly smaller (not shown) compared to untreated plants.

It can be hypothesized that a relatively high level of the JA precursor, *cis*-OPDA, contributes to the high number of de novo-growing MTs, whereas increasing levels of JA active forms (JA, JA-Ile, MeJA) after a stress stimulus (Table 1) contribute to the decreasing number and growth rate of MTs (Figure 4 and Figure 5) while triggering the stress response and inhibition of developmental processes [54]. The elevated level of *cis-*OPDA naturally occurring in *gl-1* plants can explain the thinner layers above the plasma membrane in mutant plants observed 24 h after blank treatment (Figure 6). JA signaling was described to be a repressor of cell wall damage-induced lignin production through the downregulation of cell wall modification-related genes [56,57]. Furthermore, ABA was significantly elevated in *gl-1* mutant seedlings compared to the nonstressed WT (Table 2), which could contribute to an elevated number and velocity of MTs (Figure 4 and Figure 5). However, ABA is mainly considered and discussed as a hormone involved in abiotic stress [58].

Nonstressed MTs with random organisation are sometimes bent and form large arcs (Figure 2a,b,e,f,g,m,n). MT bending was described when two MTs cross and both MTs are attached to the membrane, possibly leading to the bending of at least one of them at the crossing point [59]. Even slight changes in external conditions resulted in noticeable changes in the MT pattern. For example, treatment with the blank (enriched with salt, citric acid, and ascorbic acid compared to MS growth medium) caused enhanced MT bundling. In addition, we observed a slightly higher number of cells with more prominent waves in parallel-arranged MTs. Further remarkable progress in the bundling and wavy pattern of MT was recorded after Ag^+^ treatment.

### 3.2. Ag^+^ Treatment Led to the Organelle Appearing in the Cortical Cell Layer and a Quick Rearrangement of the MTs to Parallel Arrangement

Many cells with a parallel MT arrangement were observed, especially 1 h after Ag^+^ treatment in cotyledon cells of both lines (Figure 2 and Figure 3). In simple terms, we initially observed a rearrangement of randomly arranged MTs into parallel arrays, the bundling of MTs, and later the undulating shape of MTs appeared (Figure 2c,o and Figure 3k,o). The formation of bundles can occur with the help of e.g., crosslinking proteins from the MAP 65 (microtubule associated proteins 65) family. These proteins are able to form a bundle when the encountered MTs are at a shallow angle (below 40 degrees) to each other; such a situation is more likely when the MTs are arranged in parallel [60]. Bundles of interconnected MTs increase the stability of the microtubular network, which may be important for specific functions under stress conditions (organelle delivery, cargo, cell wall thickening) [59]. The extensive bundling of MTs is preceded by a parallel arrangement of MTs. Another severing MAP protein, KATANIN, helps to break the initial random arrangement of cortical MTs [61]. KATANIN’s severing activity can create short MTs suitable for nucleation and de novo MT growth [62,63]. This mechanism could explain the situation we observed, in which after Ag^+^ treatment the number of de novo-formed MTs gradually increased over time in both lines (Figure 4), although the growth rate of MTs varied and tended to decrease (Figure 5). At the same time, a gradually decreasing area occupied by MTs was observed (Figure S4). This phenomenon may be due to the rapid bundling of de novo-formed MTs.

Both plant lines exhibited a higher incidence of cells with wavy MTs after Ag^+^ treatment (Figure 2c,o and Figure 3k,o) and in the initial phase after NPs treatment (Figure 2d). The MT rippling phenomenon can be explained by the onset of plasmolysis caused by the presence of ions in the treatment solution. Similarly, Lang et al. described a greater extent of MT bundles, wavy MTs, as well as small bright spots between cortical MTs in the *Arabidopsis* hypocotyl during plasmolysis [64]. In fact, lower protoplast turgor caused by mild plasmolysis may lead to a decrease in MT stretch and the lateral mobility of individual MT increases, or MTs may even detach from the plasma membrane during reorganization [49].

Organelles usually present below the cortical layer in the cytoplasm may intercalate between MTs in the cortical layer, as shown in Figure 3c. Both types of organelles were visible simultaneously using the differential interference contrast technique (Figure S3). Regular black circles surrounded by a circular or irregular fluorescence signal were not identified, but we hypothesized that the 5–10 µm-long lens-shaped (fusiform) particles corresponded to endoplasmic reticulum (ER) bodies based on their shape, size, and relatively high mobility. ER bodies are organelles characteristic of many species of the order *Brassicales*, derived from the endoplasmic reticulum. In adult *Arabidopsis* plants, the presence of ER bodies is strictly restricted to roots and absent in leaves, where it can be induced de novo by wounding, herbivore attack, or jasmonic acid treatment to boost plant defense. On the other hand, ER bodies are present in all seedling organs and can move rapidly along the longitudinal axis of the cell [65,66,67]. Interestingly, we observed ER bodies less frequently in WT plants than in the *gl-1* mutant, where there were significantly higher levels of JA-Ile and JA after treatment with Ag^+^. Yamada et al. described an antagonistic effect of ethylene on JA signaling, leading to the de novo formation of ER bodies [66]. This supports our observation of ER bodies after treatment with Ag^+^. Ag^+^ inhibits the biosynthesis and perception of ethylene in plants [68]. Moreover, ethylene and its receptor ETR2 control trichome branching through MT dynamics. Trichomes do not develop in the *gl-1* mutant, and it is therefore possible that ethylene signaling is suppressed in favor of JA signaling in *gl-1* [69].

### 3.3. AgNPs Gradually Destroyed the Microtubule Dynamics and Pattern, and Substantially Altered the Hormonal Profile and Layer Thickness above the Plasma Membrane

The MT pattern of plants treated with the AgNP solution differed significantly from that treated with Ag^+^ in our experiments. AgNPs caused a relatively random pattern of less numerous MTs at most of the observed time points. Affected MTs were rodlike, relatively short filaments floating in a visible fluorescent fog with many tiny fluorescent bright spots (Figure 2 and Figure 3h,l,p). Forty-eight hours after AgNPs treatment, the dynamics of MTs were substantially decelerated, as well as the movement of supposed ER bodies, which is most likely dependent on the cytoskeleton. The fluorescent fog and bright spots are probably a mixture of oligomers of GFP-labeled tubulin, which are formed by the natural transient depolymerization of tubulin. These units subsequently bind to AgNPs and thus cannot form a new and fully functional MTs. The significant decrease in the number of MTs in our observations was accompanied by the presence of fluorescent fog. Choudhury and colleagues observed an in vitro inhibition of MT polymerization caused by citrate-coated gold nanoparticles; an AuNP could cause the aggregation of ≈10^5^ tubulin heterodimers. They explain the bond of heterodimers to AuNP due to approximately 20 free thiol groups being present on the surface of tubulin heterodimers [70]. In vitro, the interaction of the citrate-coated AgNPs and actin or tubulin was similarly observed. Using several techniques, weak binding of actin and tubulin units on the surface of AgNP, the so-called NP-corona, was confirmed; the cytoskeletal subunits showed conformational changes upon binding to AgNPs [71]. The progression of MT depolymerization and associated phenomena over time could be caused by the increasing concentration of AgNPs in the cytosol. A similar time-dependent, increasing amount of AgNPs clusters on the surface of epidermal cells was observed (Figure 1 and S2).

The dynamics of MT development after AgNPs resembled consecutive MT depolymerization more than the MT severing events followed by de novo MT growth observed after Ag^+^ treatment. The randomization of MTs followed by progressive depolymerization (lower number and growth rate of MTs—Figure 4 and Figure 5) could be the first sign of programmed cell death (PCD), which was indeed observed during our experiments in many plants 96–120 h after AgNPs treatment (not shown). A similar scenario (MT randomization → depolymerization → PCD) was observed in *Arabidopsis* after UV-B irradiation [42].

The short, less numerous, less movable and bundled MTs observed after the action of AgNPs could be of great significance for plant defence. Mirabet and colleagues link these properties of MTs to the strong anchoring of microtubules to the plasma membrane via a specific linker, such as cellulose synthase interacting protein 1 [72,73]. Cellulose synthase could contribute to the thickening of the cell wall [56]. Similarly, the motor proteins kinesins, which transport vesicles of cell wall-building material along MTs, are involved in cell wall thickening. A possible candidate in this case could be the kinesin FRA1 (fragile fiber 1), which interacts with the CMU (cellulose synthase–microtubule uncoupling) protein; the latter is able to stabilize MTs [74]. AgNPs had significantly induced a thickening of the layer above the plasma membrane in WT epidermal cells (not in mutant *gl-1* plants) 48 h after treatment (Figure 6). It is possible that the *gl-1* mutant not only has a defective kinesin encoded by the ZWI gene during trichome development [18,19,75], but also a defective FRA1 kinesin in other cells, causing the decreased deposition of material in the mutant cell wall. The plant regulates cell wall thickening and ectopic lignification in response to various stresses [56]. In *Arabidopsis* seedlings, we supposed that AgNPs could induce a response similar to microbial, mechanical, or heavy metal stress. Simultaneous stresses, e.g., mechanical or microbial, were strictly ruled out in our experiments. Internally distributed AgNPs or plant hormonal signaling must function in the thickening process in cotyledons because only plant roots were treated with the AgNP solution.

Changes in the thickness of the layer above the plasma membrane is a highly complex plant defence response that may be driven by changes in the levels of many hormones simultaneously and may very finely regulate downstream responses. Our experiments revealed a significant increase in ABA after 6 h of AgNPs treatment (the mutant had a level twice that of the WT). Seung et al. described an increase in MT dynamics from the third hour after exogenous ABA treatment and also described the reorganization of transversely arranged MTs to a longitudinal arrangement in anisotropic epidermal cells of leek (*Allium porrum* L.) leaves [76]. Additionally, in our experiments, only mutant plants exhibited a significant increase in MT growth rate between 24 and 48 h after AgNPs (Figure 5). Could this be due to a temporary increase in ABA levels? However, there was no enhancement of the layer thickness above the cytoplasmic membrane in *gl-1* mutant plants (Figure 6). Thus, it is likely that the deposition depends on other factors operating downstream of MT dynamics. In the *gl-1* mutant, only part of gibberellic or JA signaling is knocked out. An antagonistic function toward ABA upon MT reorientation has been described for gibberellic acid 3 (GA3); GA3 caused a transverse MT rearrangement and elongation of pea (*Pisum sativum* L.) internodes [77]. One possible candidate that might have reduced functionality in the *gl-1* mutant is the transmembrane integrin-like protein AT14A, which interconnects the cytoskeleton with the plasma membrane and cell wall and affects the dynamics of both types of cytoskeletal filaments in plants. In addition, the *at14a* mutant was found to have a thinner cell wall than the WT, where the gene was functional [22,78].

If we look at the stress caused by AgNPs in terms of the time course of JA derivatives levels, we find that AgNPs at some point induced signaling characteristic of mechanical stress. The *cis*-OPDA level increased 4.8-fold, JA increased 8.3-fold, and JA-Ile 16-fold in WT plants 6 h after AgNPs treatment compared to unstressed plants. In mutant plants, an increased level was also noticed (*cis*-OPDA: 1.8-fold; JA: 6.6-fold, and JA-Ile: 24.3-fold) (Table 1). According to Flokova et al., almost identical levels of JA derivatives were detected in 24-day-old untreated *Arabidopsis* WT leaves at time 0 and over 24 h, but in wounded leaves, the values varied. In addition, locally wounded leaves increased the levels of JA derivatives *cis*-OPDA and JA, similar to what we observed in our experiments after AgNP treatment, although our plants were only 6-days-old. Only the rise in JA-Ile differed in *gl-1* mutant plants after AgNP treatment (Table 1) compared to the local wounding mentioned in Flokova et al. [27]. A significantly elevated level of ABA 6 h after AgNPs more closely resembled bacterial infection [79] than wounding [27]. Although both ABA and *cis*-OPDA levels were significantly higher than in untreated plants, MTs did not further increase their dynamics; on the contrary, the growth rate of MTs was significantly reduced, as was their number (Figure 4 and Figure 5).

Taking the obtained results together, we can conclude that the levels of stress phytohormones after AgNP (with concomitant Ag^+^) treatment increased significantly (particularly in mutant *gl-1* plants) and resembled to some extent the plant response after mechanical stress. AgNP treatment reduced MT cytoskeleton dynamics (occupied area, MT number and growth rate) significantly in both plant lines at almost all observed time points. In wild-type plants, AgNPs caused an increase in the thickness of the layer above the plasma membrane after 48 h. The MT pattern in both lines after AgNPs was very different from what was observed after Ag^+^ alone or after the control. MT dynamics only differed in the plant lines in response to AgNPs by a reduced wild-type growth rate of MTs 24 h after treatment.

Ag^+^ alone caused an increased parallel arrangement and corrugation of MTs in the cotyledon cells of plants, but also an increased presence of organelles in the cortical layer of the cells.

The highest number of MTs was observed in control—unstressed plants, which may be related to the development of the first true leaves during the study; the plants treated with AgNPs or Ag^+^ developed smaller true leaves than the control. In the mutant untreated plants, the highest number of MTs was observed, but also an increased level of cis-OPDA. The development of trichomes and cell wall in wild-type plants during the above-mentioned period may, via some additional feedback signals, limit the number of MTs compared to mutant plants that do not form trichomes or reduce the MT growth rate during cell wall development after AgNP treatment.

## 4. Materials and Methods

### 4.1. Synthesis and Characterization of Nanoparticles

The monodisperse spherical (20 ± 5 nm) silver nanoparticles (AgNPs) used in this study were provided and characterized by the department of Solid State Engineering, University of Chemistry and Technology, Prague, Czech Republic, and were synthesized by a slightly adapted form of the method published by Li et al. [80]. A mixture of citrate (3 mL, 10 mg/mL), silver nitrate (0.75 mL, 10 mg/mL), and sodium chloride (3.75 mL, 187 μg/mL) was added into a boiling solution of ascorbic acid (150 μL of ascorbic acid (17.6 mg/mL) added to 142.5 mL of boiling water). The transparent and yellow solution was boiled for an hour with stirring, then left to cool to room temperature. After the synthesis, the nanoparticles were kept for 24 h in a dark place to fully finish the process of reduction and stabilization.

Prepared solutions of AgNPs were characterized by atomic absorption spectroscopy (AAS), inductively coupled plasma-mass spectroscopy (ICP-MS), and transmission electron microscopy (TEM). Concentrations of the prepared NPs were determined by means of AAS with a VarianAA880 device (Varian Inc., Palo Alto, CA, USA) using a flame atomizer at 242.8 nm wavelength. The typical uncertainty of concentration determined by this method is less than 3%.

An inductively coupled plasma-mass spectroscopy detector (ICP-MS) was used to determine the concentration of Ag^+^ originating from the unreacted silver source chemicals, using an Agilent 8800 triple-quadrupole spectrometer (Agilent Technologies, Santa Clara, CA, USA) connected to an autosampler. The AgNP colloid solution was pipetted into a 3.5 mL microtube, placed into a TLA 100.3 rotor, and centrifuged at 541,000× *g* in an Optima MAX-XP ultracentrifuge (Backman Coulter, Indianopolis, IN, USA) for 0.5 h. After this, 1.0 mL of the supernatant was carefully removed using a pipette and ICP-MS analyzed. Sample nebulization was performed using a MicroMist device equipped with a peristaltic pump. Pure buffer solution (2.2 mM sodium citrate) was used as a blank sample. The uncertainty of the measurement was less than 3%.

TEM images were taken using a JEOL JEM-1010 (JEOL Ltd., Tokyo, Japan) operated at 400 kV. The colloidal solution was placed on a copper grid coated with a thin layer of amorphous carbon film on a filter paper. The excess solvent was removed. Samples were air-dried and kept under vacuum in a desiccator before placing them on a specimen holder. Particle size was measured from the TEM micrographs and calculated by taking into account at least 500 particles.

### 4.2. Preparation of Silver Nanoparticles for Experiments

The initial concentration of AgNPs in aqueous stabilization buffer (0.8 mM NaCL, 0.1 mM ascorbic acid, 0.7 mM sodium citrate, pH 6.8) was around 30 mg/L and then further concentrated by centrifugation at 5000× *g* for 30 min and 15 °C and diluted to the required stock concentration of 450 mg/L with sterile stabilizing buffer. The concentration of this working solution was further checked by spectrophotometry at 401 nm and AAS.

To reach the final AgNPs working concentration of 150 mg/L, the stock solution had been diluted just *ex tempore* by the addition of 1/16 MS medium—1:2).1/16 MS medium was used instead of the commonly used 1/2 MS to reduce the naturally occurring clustering of NPs into NP aggregates caused mainly by phosphate or sulphate anions 1/16 MS medium was determined to be the appropriate concentration of plant nutrients with a minimal clustering effect on AgNPs, and was used throughout the experimental design as a growth medium and as one of the control treatments. To evaluate the effect of NP stabilizing buffer present in AgNPs solution, treatment with a mixture of stabilizing buffer and 1/16 MS for nanoparticles (1:2) was further included throughout the experiment design as a control, and this experimental variant is referred to as ”blank“. In the 150 mg/L AgNPs working solution, free Ag^+^ were determined in the range of 51 to 52 μg/L. Hereafter, treatment with free Ag^+^ in the concentration of 50 μg/L (10mg/L AgNO_3_ stock solution diluted with 1/16 MS medium) was included throughout the experimental design as an additional control for the effect of AgNPs.

### 4.3. Plant Material

#### 4.3.1. Plant Cultivation for Microtubular Dynamics Experiments

The experiments were performed using 7- and 5-day-old seedlings of the wild-type (WT) and *gl-1* (GLABRA 1) mutant of *Arabidopsis thaliana* (ecotype Columbia-0), both with fluorescently labeled microtubules (GFP-TUA6; GFP-tagged tubulin α 6), hereafter referred to as WT and *gl-1*, respectively. The WT line was obtained from Dr. Chris Ambrose at the University of British Columbia, Canada (made according to [81]). The *gl-1* mutant was obtained from Professor Hashimoto at the Nara Institute of Science and Technology, Japan [82].

Seeds were sown under sterile conditions on agar plates containing 1/16 MS medium [83]; 1.2% plant agar and 1% *w*/*v* sucrose). After 2 days of cold stratification, seedlings were grown on vertically oriented plates in a climate chamber at 22 °C, 50% air humidity, under long day conditions (12 h light period) with light intensity 120 μmol m^−2^ s^−2^. Seedlings were transferred into the above-mentioned solutions for treatment on the five or seventh day of growth.

#### 4.3.2. Modified Cultivation for Auramine O-Stained Layer Observation and Phytohormone Analysis

To only treat the roots and the lower part of the hypocotyl, plants were cultivated in the space created between the two slides, the size of the gap (about 0.5 mm) between the slides was adjusted by inserting a strip of parafilm between the two shorter edges of the slides. The slides were then placed vertically on a resealable glass dish containing 15 mL of 1/16 MS growth medium; the medium rose by capillary action between the slides to the top edge. Seeds were placed in the gap at the top of both slides at 7-mm intervals, and roots were grown through the solution in the gap between the two microscope slides. The glass dish was closed, sealed with parafilm, and after two days of cold stratification, left in the climate chamber as above until the fifth day. Then, the growth medium was changed to the individual treatment solutions without handling the plants under sterile conditions, the trays were sealed and placed again in the growth chamber with the above parameters for another 6 h (for phytohormone analysis) or 24, 48, and 72 h (for observation of the Auramine O-stained layer). Plants for phytohormone analysis were immediately frozen in liquid nitrogen after 6 h of treatment and stored at −80 °C.

### 4.4. Modified FRAP Method

**Figure 7:**
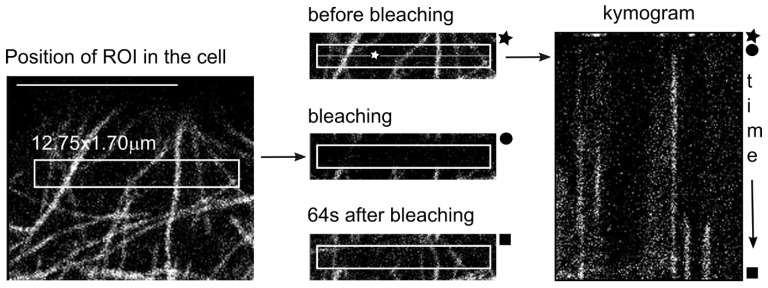
Modified FRAP method. The left image depicts cortical microtubules in a cotyledon epidermal cell of *Arabidopsis thaliana* WT expressing GFP-TUA6 construct for MT visualization. The experiment proceeded in the rectangle—ROI (region of interest). The three images in the middle demonstrate the situation in the ROI during the FRAP experiment: in the upper picture are MTs before GFP destruction, below are MTs immediately after bleaching with the high intensity of the laser beam, and the third picture shows MTs which appeared de novo within 64 sec of scanning. On average, 200 images were taken. The white asterisk indicates the line where the light intensity of de novo-formed MTs was monitored during the experiment. Chronologically ordered fluorescence intensities along the middle line from each image of the time stack were collected into a single image called a kymogram—on the right. The position, which equates to the time at which a particular MT reached the midline, is clearly visible here. The black asterisk, circle, and square on the kymogram indicate the time corresponding to the situation of the three frames in the middle of the picture. Images were taken with a Zeiss 880 CLSM. Scale bar = 10 µm.

A new microscopic method based on the FRAP technique (Fluorescence Recovery After Photobleaching), was developed to assess MT dynamics. Images of the microtubular region in cotyledon epidermal cells were acquired with Zeiss 880 confocal laser scanning microscope with a 63x Plan-Apochromat Oil DIC M27 (NA 1,4) objective. Ex./Em.: 488/499–561 nm. Image resolution was 792 × 792 px, pixel dwell: 0.42 µs (cca 320 ms per image), pinhole setting: 1 AU (optimal axial resolution: 380 nm). Up to five regions of interest (ROIs) with a fixed size of 150 × 20 px (12.75 × 1.70 µm) were set up in each cell, and the experiment was performed there (Figure 7). After the first three frames, the ROIs were bleached with a high intensity laser beam and then a time stack of 70 s was taken with approximately 200 frames, in which microtubules gradually grew de novo in the ROI area. The time stacks were processed using the Software Image J: a line was constructed down the middle of the each ROI of the time stack (Figure 7). MTs emerging de novo in the bleached area can reach the middle line after an 850-nm-long trajectory. Chronologically ordered middle lines were collected into one image called a kymogram (Figure 7), and the position corresponding to the time when a particular MT reached the middle line was clearly visible. When there were faint or unclear lines in the kymogram, the dynamics of MT was compared with the time-stack data to avoid artifacts. The de novo MT growth rate (nm per second) was calculated from the data. In each ROI, the number of de novo emerged MT was counted. Afterwards, using the same threshold, the kymogram pictures were transformed into binary images and in the area of the three first frames (before bleaching), the percentage of white pixels (position of MTs) was measured.

### 4.5. Technical Notes on Confocal Microscopy—Imaging of MT

The width of the MT is 25 nm; this is below the lateral resolution limit of the confocal microscope used (203 nm according to the Rayleigh criterion). Therefore, we were unable to distinguish whether a bundle of, e.g., 4 or 7 individual MTs was being observed. When the MTs are lined up next to each other (which is unlikely), the point spread function of a 100 or 175 nm spot will be imaged as a region of very similar size, but the GFP signal intensity is expected to be higher for a bundle with a larger number of MTs below the resolution limit than for a bundle with fewer MTs. Thus, it is quite difficult to distinguish whether the lower MT occupancy was due to fewer MTs or due to the bundling of several MTs together and vice versa. To assess this uncertainty, we also monitored the number of MTs growing after photobleaching in the same region where the MT occupancy percentage was measured a few seconds earlier (Figure 4 and S4). We also noted bundles of MTs with higher fluorescence intensity in the Section 2.

### 4.6. Microscopy and Image Processing for Auamine O Stained Layers

Treated seedlings of GFP-TUA6 tagged wild-type or *gl-1*mutant plants, were stained one by one immediately before microscopy as follows: individual plants were placed at the microtube filled with 0.01% Auramine O solution for 10 min (Sigma-Aldrich; 0.1 *w*/*v* in 0.05M Tris-HCl, pH 7.2 stock solution diluted with 1/16 MS growth medium to final concentration), and were afterwards transferred onto a microscopic slide.

Images were acquired with the CLSM 880 inverted confocal microscope and CLSM 880 confocal microscope in a horizontal setup with a vertical stage (Carl Zeiss, Inc., Jena, Germany) equipped with a Plan-Apochromat 63x/1.40 Oil DIC M27 objective and with a 458 nm argon laser used for excitation. Emitted light was captured using a MBS 458/561 beam splitter and a 491–571 nm band-pass filter. Pinhole setting: 1 AU. To determine the thickness of the fluorescently labeled layers, optical sections were taken with an optimal size of 372 nm in the Z plane. The Z stack typically contained 5 to 10 frames. The first image was taken at the epidermal cell surface as soon as the fluorescence signal was detected, and the last image was taken immediately after the fluorescence signal had declined and the cortical layer of GFP-labeled microtubules below the plasma membrane was reached. The thickness of Auramine O-stained layer outside of the plasma membrane was determined in Image J by the number of optical sections with Auramine O staining present and simultaneously using the “profile” tool in ortho projection of the image.

### 4.7. Analysis of Phytohormones

Endogenous levels of jasmonates (jasmonic acid, JA; salicylic acid, SA; and abscisic acid, ABA) were determined in 10 mg (around 50 seedlings) of plant material according to the method described by Flokova et al. [27]. All experiments were repeated as four biological replicates. Briefly, the phytohormones were extracted using 10% methanol with a cocktail of stable isotope-labelled standards added as follows: 10 pmol of [^2^H_6_] JA, [^2^H_6_] ABA, and 20 pmol of [^2^H_4_] SA (all from Olchemim Ltd., Olomouc, Czech Republic) per sample. The extracts were purified using Oasis HLB columns (30 mg/L ml, Waters) and then evaporated to dryness under a stream of nitrogen. Jasmonate levels were quantified by ultra-high performance liquid chromatography-electrospray tandem mass spectrometry (an Acquity UPLC I-Class System coupled to a Xevo TQ-S MS, all from Waters) using stable isotope-labelled internal standards as a reference.

### 4.8. Statistical Procedures and Plot Design

Statistical significance was tested via one-way or multifactorial ANOVA followed by Tukey’s post-hoc HSD test. When normality and/or homoskedasticity (the Shapiro-Wilks’s test) were violated (typically phytohormone concentrations), log-transformation highly improved the distribution (Gaussianity) and those log-transformed data were tested.

MT dynamics data were processed and plots created using the RStudio development environment for the R programming language and open-source packages (e.g., ggplot2 and tidyverse).

## Figures and Tables

**Table 1:** Fresh weight-based concentrations of jasmonic acid derivatives in treated 5-day-old seedlings.

	Line	Blank	Ag^+^	AgNPs
		pmol g^−1^ FW (mean ± StD)	pmol g^−1^ FW (mean ± StD)	pmol g^−1^ FW (mean ± StD)
*cis*-OPDA	WT	32,560 ± 8007 b	153,900 ± 8903 a	158,100 ± 4047 a
	*gl-1*	80,580 ± 3976 c	38,820 ± 10,080 b	145,600 ± 17,760 a
JA	WT	191± 24 d	364 ± 26 ef	1575 ± 55 g
	*gl-1*	274 ± 48 e	482 ± 97 f	1802 ± 415 g
JA-Ile	WT	14.9 ± 2.5 h	18.3 ± 0.6 h	240 ± 22 j
	*gl-1*	18.9 ± 1.5 h	31.3 ± 2.6 i	458 ± 60 k
9,10-DHJA	WT	435 ± 133 lo	721 ± 172 lm	766 ± 86 lm
	*gl-1*	267 ± 79 n	200 ± 37 n	1242 ± 416 m
11,12-OH-JA	WT	1140 ± 168 q	2400 ± 381 q	10,730 ± 1013 r
	*gl-1*	2653 ± 730 q	1569 ± 259 qp	15,230 ± 4360 r

*cis*-12-oxo-phytodienoic acid (*cis-*OPDA), jasmonic acid (JA), (−)-jasmonoyl-L-isoleucine, (JA-Ile), (±)-9,10-dihydrojasmonic acid (9,10-DHJA) and sum of (±)-11 and (±)12-hydroxyjasmonic acid (11/12-OH-JA) determined with UHPLC-MS/MS in WT and *gl-1* lines of *A. thaliana* plants after 6 h of control (blank), Ag^+^ (50 µg/L), and AgNPs (150 mg/L) treatments. Significance is determined by bidirectional ANOVA and the Tukey test. Different letters denote significant differences at *p* < 0.05, *n* = 4.

**Table 2:** Fresh weight-based concentrations of abscisic acid derivatives and salicylic acid in treated 5-day-old seedlings.

	Line	Blank	Ag^+^	AgNPs
		pmol g^−1^ FW (mean ± StD)	pmol g^−1^ FW (mean ± StD)	pmol g^−1^ FW (mean ± StD)
ABA	WT	3.88 ± 0.4 a	6.55 ± 0.6 a	51.2 ± 2.1 c
	*gl-1*	7.92 ± 0.7 b	6.78 ± 1.0 a	115.0 ± 13.0 d
DPA	WT	ND	ND	270 ± 18 e
	*gl-1*	ND	ND	630 ± 136 f
PA	WT	ND	ND	283 ± 17 g
	*gl-1*	ND	ND	374 ± 42 g
SA	WT	1957 ± 293 h	2124 ± 412 hi	2857 ± 184 i
	*gl-1*	1560 ± 192 h	971 ± 162 j	5032 ± 535 k

abscisic acid (ABA), dihydrophaseic acid (PDA), phaseic acid (PA), and salicylic acid (SA), determined with UHPLC-MS/MS in WT and *gl-1* lines of *A. thaliana* plants after 6 h of control (blank), Ag^+^ (50 µg/L) and AgNPs, 150 mg/L treatments. Significance is determined by bidirectional ANOVA and the Tukey test. Different letters denote significant differences at *p* < 0.05, *n* = 4.

## Data Availability

Data are available from the corresponding author (jindriska.angelini@vscht.cz (accessed on: 14 July 2021) at reasonable request.

## Supplementary Materials

The following supporting information can be downloaded at: https://www.mdpi.com/article/10.3390/plants11030313/s1, Figure S1: Cotyledon surface in different nontreated *Arabidopsis thaliana* lines; Figure S2: Direct visualization of clumps in hypocotyl cells after AgNPs treatment; Figure S3: Presence of organelles in the cortical layer of MT after treatment with Ag^+^ ; Figure S4: Percentage of area occupied by microtubules in epidermal cells of the cotyledon.
