# Supplementary material for: Silver Nanoparticles Alter Microtubule Arrangement, Dynamics and Stress Phytohormone Levels
Source: Plants (Basel). 2022 Jan 25;11(3):313. doi: 10.3390/plants11030313 (PMC8838976; doi:10.3390/plants11030313)
Supplement: Supplementary file 1 [file plants-11-00313-s001.zip › plants-1361099-supplementary.pdf]

Supplementary material:

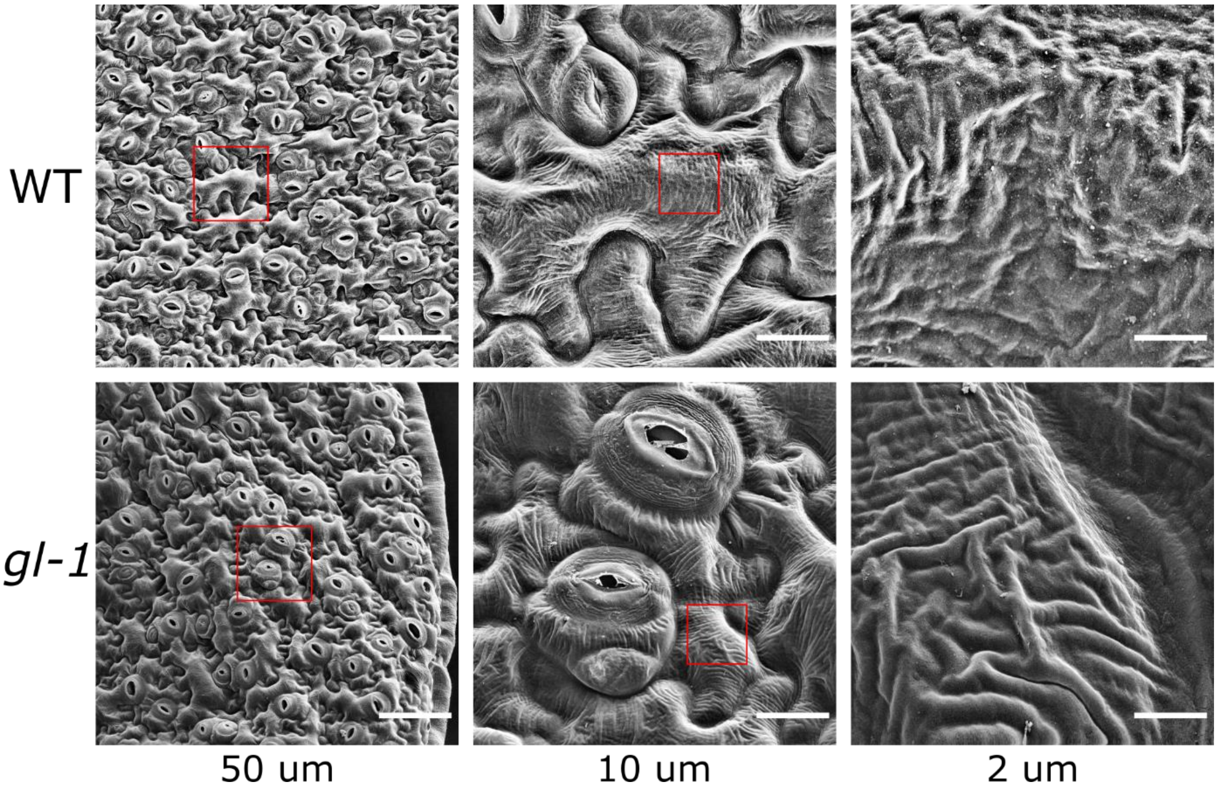

Figure S1: Cotyledon surface in different nontreated *Arabidopsis thaliana* lines

Scanning electron micrographs of the abaxial epidermis 10-day-old wild-type (WT) and *gl-1* mutant cotyledons. The preparation of plants for microscopy was carried out simultaneously for all plants. Images were obtained with Lyra3 GMU FIB-SEM. The scale size is indicated below each column of figures.

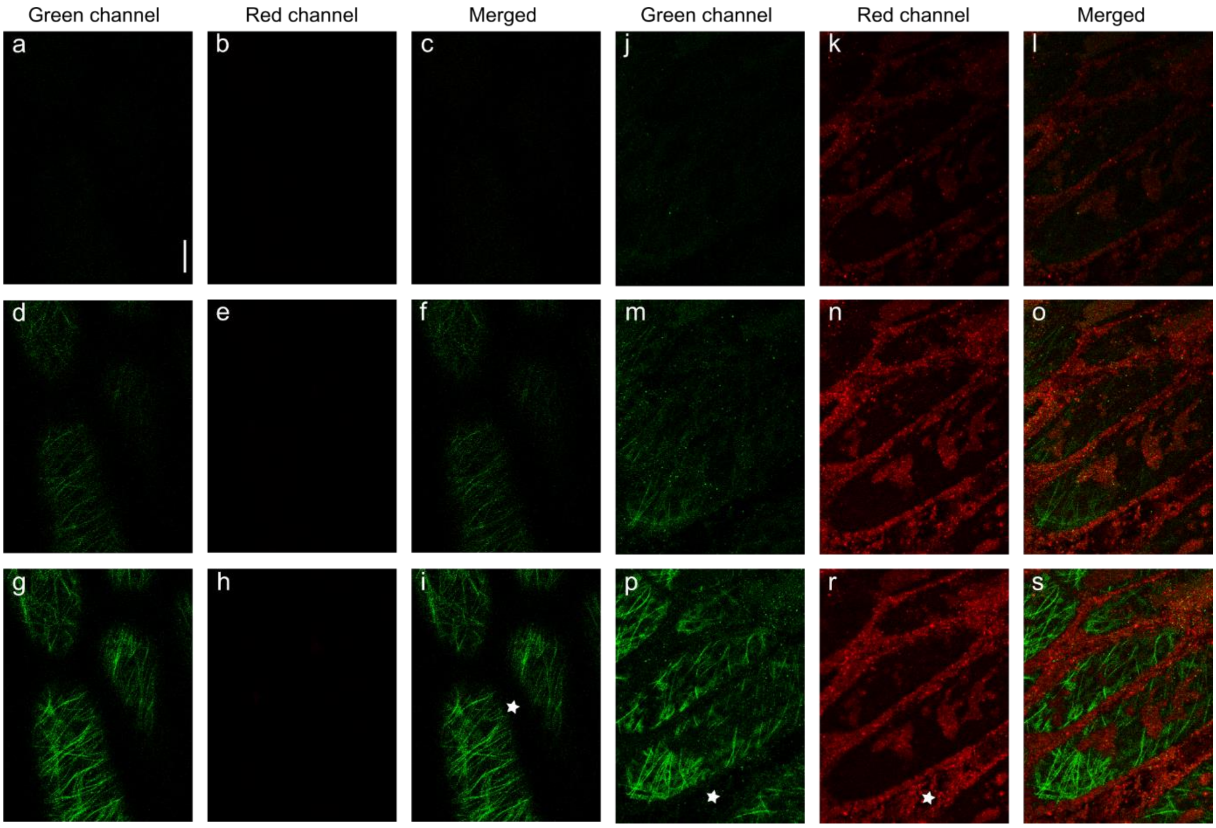

Figure S2: Direct visualization of clumps in hypocotyl cells after AgNPs treatment
Arabidopsis plants (5-day-old) with GFP-tagged microtubules (GFP-TUA6 in WT background) were treated for 24 h with growth medium (**a**-**i**) or 150 mg/l AgNPs solution (j-s). Images were taken sequentially with a Zeiss 880 CLSM; the images: (**a**,**d**,**g**,**j**,**m**,**p**) in the green channel (ex/em: 488/496-556 nm) or in the red channel (ex/em: 561/563-652 nm) - (**b**,**e**,**h**,**k**,**n**,**r**); the remaining pictures are the merge of both channels. The first row of images focus on the surface of epidermal cells in the upper part of the hypocotyl, images (**k**) and (**l**) show clusters of AgNPs on the surface of the cell wall. The second row of pictures shows the optical layer 1 μm below the first – in the cell wall or instantly below the plasma membrane; the green spots in the images m and o represent clumps of free tubulin units tagged with GFP. The third row of pictures shows the optical layer 1 μm beneath the second layer – inside the microtubular layer. The white asterisks in images (**i**,**p**,**r**) indicate grooves between cells, where AgNPs have accumulated (**r**,**s**). Scale bar = 10 μm.

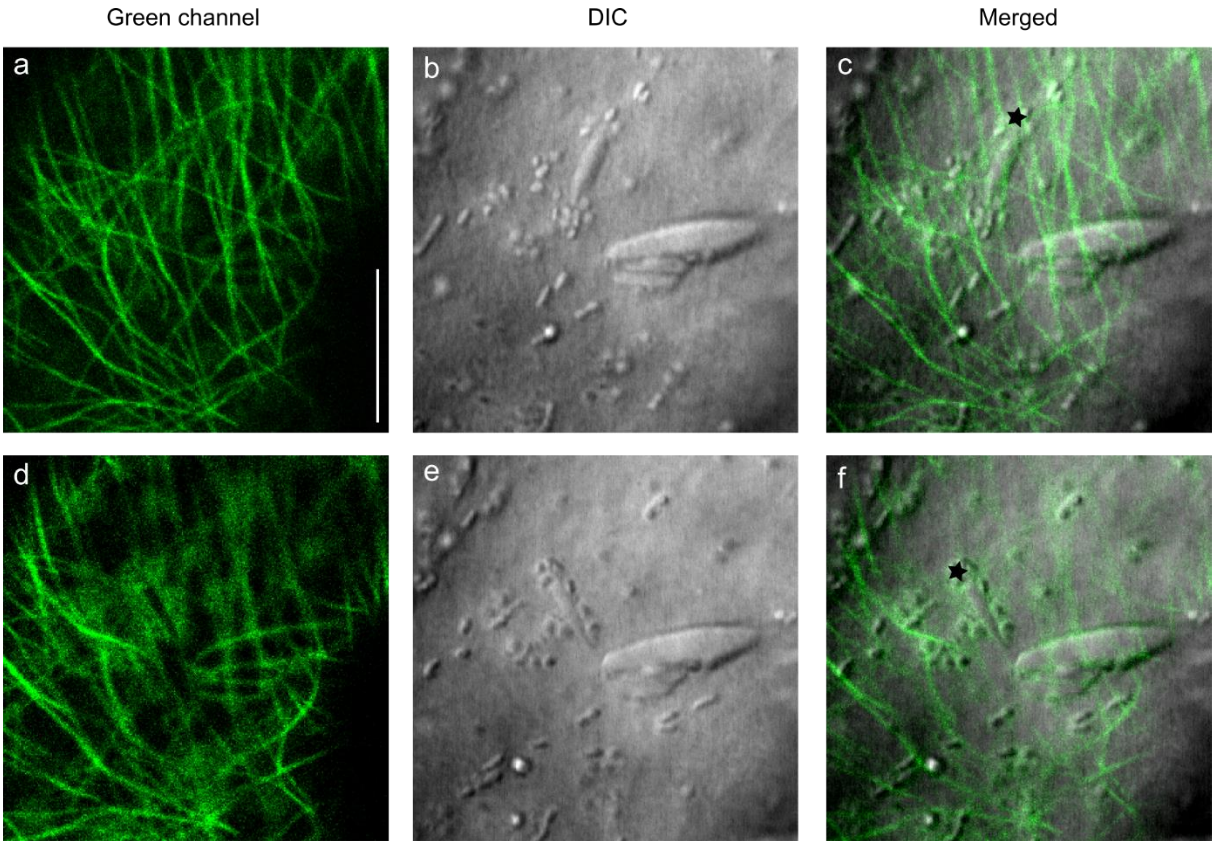

Figure S3: Presence of organelles in the cortical layer of MT after treatment with Ag^+
Seven-day-old *Arabidopsis* plants with GFP-tagged MT (GFP-TUA6 in *gl-1* background) were treated with 50 µg/l Ag^+ solution for less than 1 h. The images of cotyledon cell were taken with a Zeiss 880 CLSM in the green channel (ex/em: 488/496-556 nm) - (**a**,**d**). Or with thedifferential interference contrast (DIC) technique - (**b**,**e**); the pictures (**c**,**f**) are the merge of both channels. The first row of images shows the identical spot in the cortical layer of MT, 760 nm below the plasma membrane (below the beginning of the MT layer). The lower row of images lies 1 150 nm below the plasma membrane, and it is imaged 0.83 s later than the first row of images. The black asterisks indicate one of the lens-shaped organelles, probably the ER body. Its position in the first and second rows of images shows the very fast movement of some of these organelles. Scale bar = 10 µm.

The values of the area occupied by the MTs had a predominantly inverse trend over time compared to the number of MTs that appeared *de novo*.

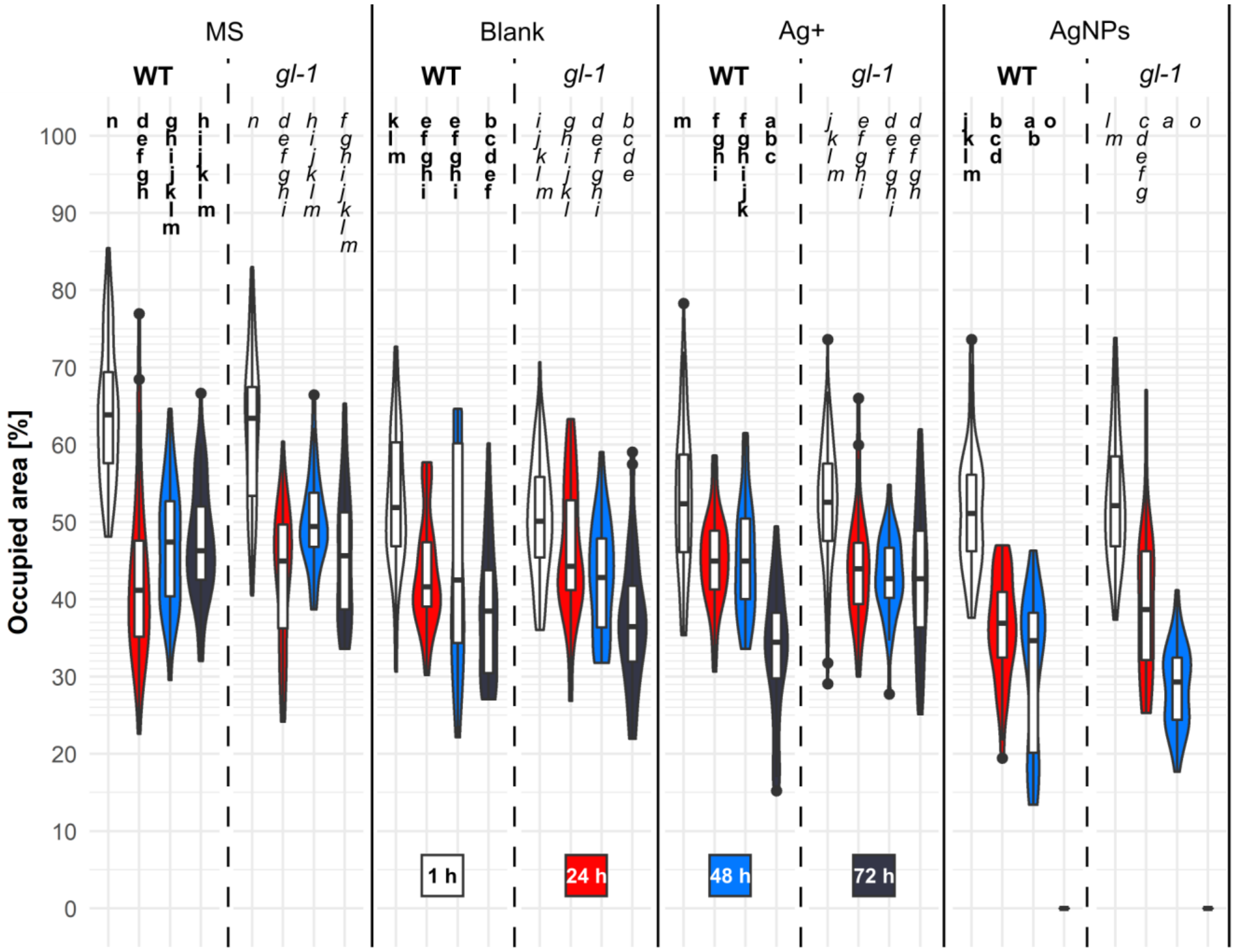

Figure S4: Percentage of area occupied by microtubules in epidermal cells of the cotyledon. Seven-day-old *Arabidopsis* plants (wild type or mutant *gl-1*) were treated for 1, 24, 48 or 72 hours with growth medium (MS), nanoparticle stabilizing buffer (Blank), 50 µg/l of silver ions (Ag+) or 150 mg/l of silver nanoparticles (AgNPs). For one cell, usually up to five regions with the same size (3.25 μm^2) were chosen in which the area occupied by MTs was measured a few seconds before bleaching. Images of one optical section (optimal axial resolution: 380 nm) were acquired with a Zeiss 880 CLSM and analyzed with the software ImageJ. Values represent averages of the area occupied by MT ± SD shown by white boxes inside the area of the violin plot (n = 35-100). The width of the violin plot represents the number of observations seen for the individual percentage of the MT area. Significance is determined by multifactorial ANOVA and the post hoc unequal Tukey test. Different letters denote significant differences at $P < 0.05$.
